# New Insights into Green Protocols for Oxidative Depolymerization of Lignin and Lignin Model Compounds

**DOI:** 10.3390/ijms23084378

**Published:** 2022-04-15

**Authors:** Cecilia Scimmi, Luca Sancineto, Jozef Drabowicz, Claudio Santi

**Affiliations:** 1 Group of Catalysis Synthesis and Organic Green Chemistry, Department of Pharmaceutical Sciences, University of Perugia, Via del Liceo 1, 06122 Perugia, Italy; cecilia.scimmi@studenti.unipg.it (C.S.); luca.sancineto@unipg.it (L.S.); 2 Institute of Chemistry, Jan Dlugosz University in Czestochowa, Armii Krajowej 13/15, 42-200 Czestochowa, Poland; j.drabowicz@ujd.edu; 3 Division of Organic Chemistry, Center of Molecular and Macromolecular Studies, Polish Academy of Sciences, Sienkiewicza 112, 90-363 Lodz, Poland

**Keywords:** lignin, oxidation, green chemistry, biomass, model compounds

## Abstract

Oxidative depolymerization of lignin is a hot topic in the field of biomass valorization. The most recent and green procedures have been herein detailed. Photochemical and electrochemical approaches are reviewed highlighting the pros and cons of each method. Mechanochemistry activated strategies are able to combine oxidation and depolymerization in the deconstruction of lignin. Homogenous and heterogeneous catalytic systems are exemplified stressing the green aspects associated with both the procedures. Solvent-free approaches as well as those carried out in alternative media are listed. Finally, the few examples of selenium catalyzed lignin valorization reported so far are cited.

## 1. Introduction

**Figure 1:**
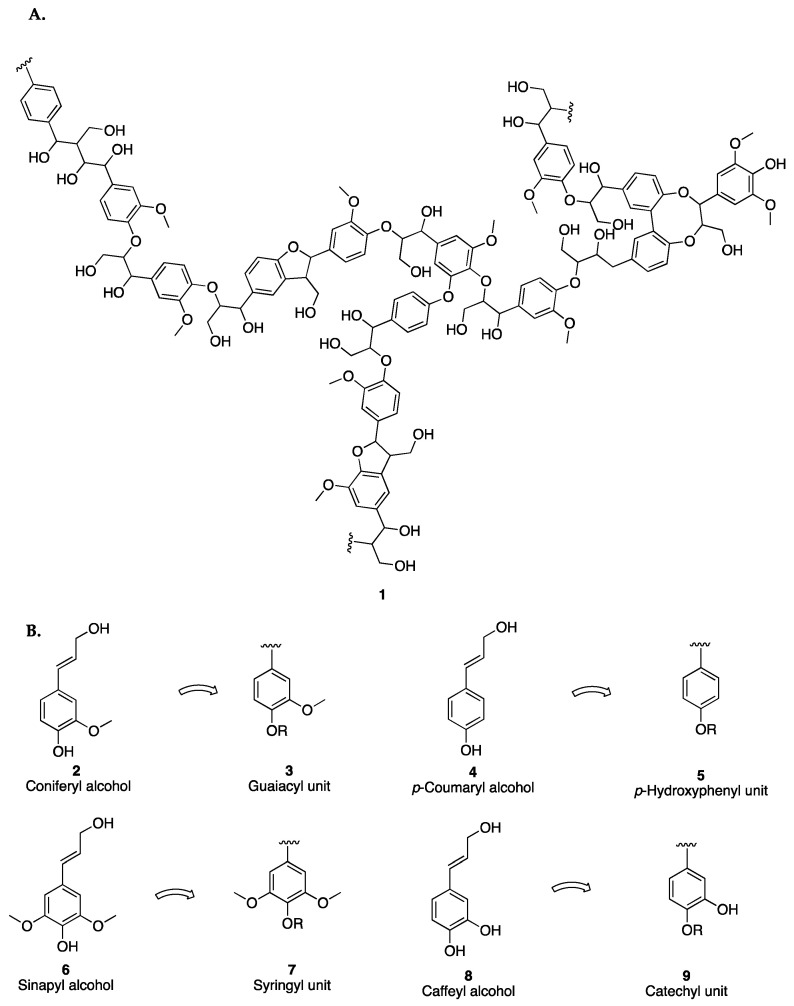
(**A**) Lignin structure; (**B**) monomers and monolignols.

Lignocellulosic biomass is one of the most abundant materials on earth and represents a major source of non-fossil carbon [1,2]. It is composed by three biopolymers: lignin (Figure 1A), cellulose and hemicellulose [3]. All of these are components of plant cell walls and lignin confers key properties such as rigidity, resistance against pests and pathogens, and is also essential for mineral transport [4,5]. Lignin biosynthesis starts in the cytoplasm where the three main monomers, sinapyl alcohol, coniferyl alcohol and p-coumaryl alcohol, are prepared (Figure 1B) [6]. They are successively transferred into the apoplast where the last step is carried out by two enzymes, peroxidase and laccase. It consists of the polymerization of the three monomers that are converted into the corresponding monolignols called p-hydroxyphenyl, guaiacyl and syringyl units [7] (Figure 1B). Recently, caffeyl alcohol was also identified as an unusual monolignol (C-monolignol) in the vanillin seeds [8] (Figure 1B). Monolignols are distributed in lignin in a varied way, that, for this reason, assumes a variable structural composition which depends on many factors such as the plant’s age and species [9]. For example, hardwood lignin is principally composed of sinapyl and coniferyl alcohol whereas in softwood the most abundant monomer in lignin is p-coumaryl alcohol [10]. The aromatic units of lignin are linked by linkages that are divided in two groups: C-C bond (β-5, β-β, β-1, 5-5) and the more abundant C-O bond (β-O-4, α-O-4, α-O-γ, 4-O-5) [11].

Industrially, lignin is waste from the pulp and paper industry that generates 50 million tons yearly as a side product known “black liquor” [12]. This huge amount of byproduct can serve as a source of energy. Indeed in a world with a continuously increasing energy demand and high energy costs [13], black liquor can be considered a good, clean and renewable alternative to fossil fuels [14]. As a result, lignin undergoes different treatments such as gasification [15], pyrolysis and combustion [16] to generate gas and heat. On the other hand, lignin could be treated to obtain valuable products such as materials (e.g., hydrogel and 3D printing lignin−plastic composites) [17] and fine chemicals [18] for the pharmaceutical, cosmetic and food industries [19]. 

Lignin has to be depolymerized to retrieve fine chemicals and the most diffuse depolymerization processes are represented by hydrogenolysis and oxidation [20]. Oxidation is the favorite method because it is often performed under mild conditions leading to cleaner end products [21]. The monomeric products that generally are isolated from the oxidative depolymerization of lignin are both aromatic compounds—such as aldehydes, acids and ketones—and aliphatic compounds [22]. 

The most commonly used methods in the lignin oxidation are performed with chlorine and nitrate, both of which are non-eco-friendly, toxic and polluting oxidants [21]. The need to develop greener depolymerization processes has led to the optimization of new oxidative methods [21]. In these protocols oxidants are replaced by hydrogen peroxide [23] and molecular oxygen [24]. Moreover, in respect to the principles of the green chemistry [25,26], a large part of these reactions are performed in the presence of catalysts, with a particular interest for the heterogeneous ones characterized by an improved recoverability and reusability, reducing the waste-production at the end of the process [27]. Similarly, non-green solvents, when possible, were substituted with water [28] or other eco-friendly media [29]. Non-conventional activation methods such as electrochemistry and photochemistry were also applied to the lignin oxidation [30]. 

Due to the large and growing interest in this topic, various reviews have appeared in recent years. Among the most recent, in 2019 Teong et al. provided a series of examples where lignin was oxidized with hydrogen peroxide [23]. In 2021 another article reviewed all the eco-friendly protocols applied for conversion of lignin into fine chemicals and fuels [31]. 

Among the most representative methods worth mentioning are those reported by Mottweiler and coworkers [32] that performed a successful oxidation of lignin using Fe-DABCO as catalyst combined with hydrogen peroxide; Crestini et al. [33,34] conducted oxidation using methyltrioxorenium(VII) (MTO, CH_3_ReO_3_) and four different heterogenous derivatives of MTO starting from hydrolytic sugar cane lignin, red spruce kraft lignin and hardwood organo-solvent lignin. The oxidations performed using polyoxometalates (POMs) as catalysts are considered green reactions [35] and one of the most efficient oxidation treatment of lignin carried out with these catalysts was developed by Voitl et al. [36] that tested the activity of H_3_PMo_12_O_40_. Very interesting are also the studies reported by Badamali et al. [37,38] where Co-salen complexes were used as catalysts. In particular, a heterogenous Co-salen complex called ([N,N’-bis(salicylidene)ethane-1,2-diaminato]Cobalt(II) immobilized on SBA-15 (Santa Barbara Mesoporous silica) was used and the oxidation was mediated by H_2_O_2_ under microwave irradiation as an alternative green energy source [39].

As this field is constantly developing, we here present an updated state of the art review focused on the most recent and sustainable protocols developed for lignin oxidation.

## 2. Novel Methods for the Green Oxidative Depolymerization of Lignin

### 2.1. Photochemical and Electrochemical Approaches

**Scheme 1:**
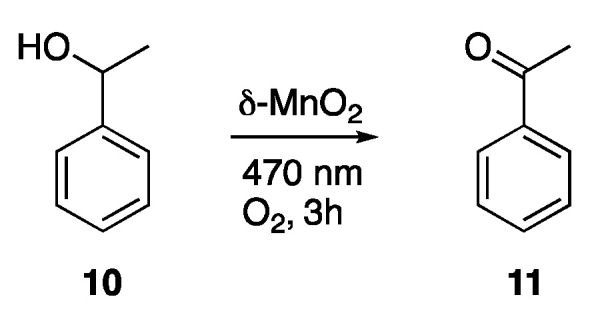
Oxidation of **10** under optimized condition.

In recent years, several studies have been carried out on eco-friendly lignin oxidative depolymerization using photochemistry. This approach can be considered green as it can be theoretically envisioned the use of solar radiation as a renewable and non-polluting energy source [30]. One of the most recent and sustainable applications of photocatalysis was developed by Dai et al. [40]. In this work, lignin was oxidized by irradiation with different wavelengths using MnO_2_ as a catalyst and molecular oxygen as an oxidant. The reactions were initially performed starting with 1-phenylethanol (**10**) as a lignin model compound that was oxidized into the corresponding ketone (**11**) (Scheme 1). Different amounts of MnO_2_ were checked under UV and blue LED irradiation demonstrating that the final yields are directly proportional to the amount of catalyst. Moreover, the activity of the catalyst was tested screening α, β, γ and δ-manganese dioxide, and the δ-MnO_2_ turned to be the best-in-class since it can be recovered by treatment in a furnace at 230 °C for 15 min in air and reused without any loss in catalytic efficiency.

**Figure 2:**
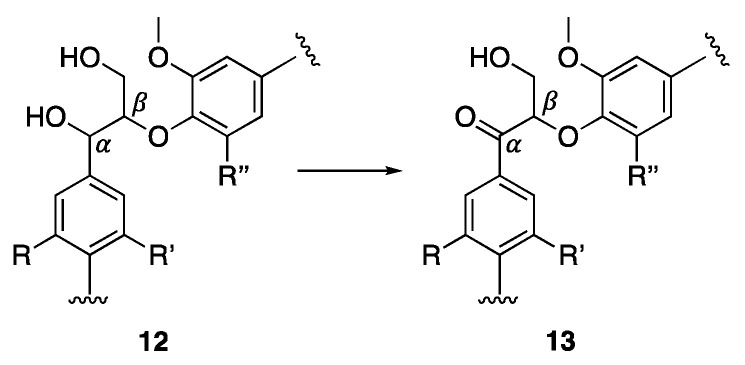
Lignin samples oxidation.

The optimized conditions (10 mL of acetonitrile, 870 mg of MnO_2_, 3 h with oxygen bubbled under blue light irradiation = 470 nm) were applied on 200 mg of lignin samples (kraft, organosolv, alkali lignin). The analyses performed by IR showed an increase in the bands of the C=O group while from the 2D-NMR spectrum the conversion of the benzylic alcohol (α-position) into the ketone was observed, together with a downfield shift of the proton at the β-position (Figure 2). 

The depolymerization process was performed by treating oxidized lignin samples with 2-hydroxy ethylammonium formate. This treatment afforded a material with a strongly reduced molecular weight (from 1400 to 400) [41]. 

**Scheme 2:**
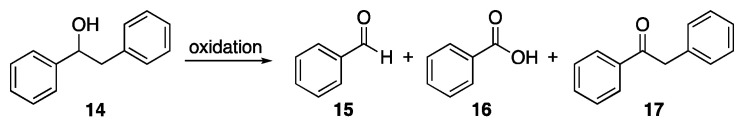
β-1 lignin model compound oxidation.

Another example of photocatalysis applied to lignin depolymerization was reported by Wu et al. [42]. In 2021 they developed a Z-scheme Ag_3_PO_4_-polymer carbon nitride nanocomposite that was tested in the oxidative cleavage of C-C bonds. Initially polymer carbon nitride (PCN) and other semiconductors (TiO_2_, ZnO, BiVO_4_, Cu_2_O, CeO_2_, BiOCl, BiOI and CdS) were screened for the oxidation of 1,2-diphenyl ethanol (**14**) as a β-1 lignin model compound (Scheme 2). 

**Scheme 3:**
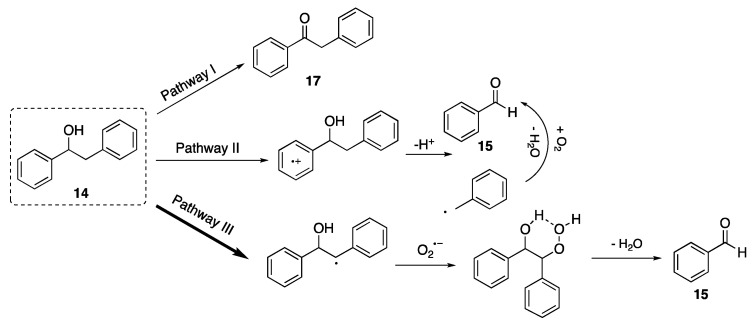
Oxidation mechanisms.

PCN was selected as the best catalyst reaching, after 6 h, 15% yield with benzaldehyde (**15**) as the main product with a chemoselectivity of 68%. Successively, different heterojunctions (10Ag_3_PO_4_-90PCN, 20Ag_3_PO_4_-80PCN, 40Ag_3_PO_4_–60PCN, 60Ag_3_PO_4_–40PCN and 80Ag_3_PO_4_–20PCN) were synthesized with the goal to increase the photocatalytic activity of PCN. Among them, 40Ag_3_PO_4_-60PCN showed the best catalytic performance affording **17** and benzaldehyde in 86%yield, without the formation of benzoic acid (**16**). Three different pathways were hypothesized to be involved in the oxidation mechanism. The first is the oxidation of benzylic alcohol **14** into the corresponding ketone **17**, the second involves the C-C bond cleavage through the formation of a radical cation with the final formation of **15**. The third pathway, that was supposed to be the major one, consists of the formation of a beta radical that successively interacts with superoxoradical creating a six membered transitional state that undergoes intramolecular electron transfer resulting in the C-C bond cleavage affording **15** (Scheme 3). Finally, this protocol was successfully applied to different lignin β-1 model compounds and on lignin samples in the presence of O_2_ and using a 300 W xenon lamp with AM 1.5 filter as a light source. When the z-scheme was tested on the raw lignin sample, the formation of aromatic aldehydes and acid monomers was observed confirming that this method is suitable for the cleavage of the C-C bonds present in lignin. 

**Scheme 4:**
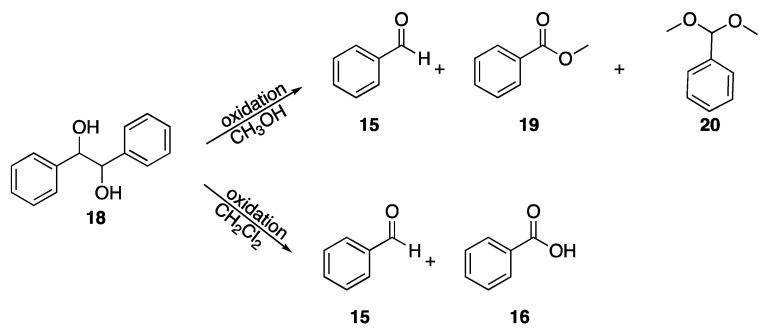
C-C bonds cleavage products in methanol and dichloromethane.

Another very recent case of oxidation of C-C bonds was reported by Yang et al. [43]. They reported a green alternative to the classic method that use stochiometric amounts of chromium trioxide. Essentially, this is a photocatalytic oxidation of 1,2 diols used as model compounds, and is based on the use of molecular oxygen as oxidant in the presence of various vanadium based photocatalysts. At first, a series of experiments using hydrobenzoin (**18**) as a substrate were performed adopting the following conditions: 0.1 g of substrate, 0.05 g of a catalyst in 10 mL of a solvent under 0.2 Mpa O_2_, Xe lamp with a light intensity of 220 mW cm^−2^, r.t. for 5 h. Intriguingly, different products were obtained depending on both the solvent and photocatalyst used in the reaction. When methanol was used, three main products were identified: benzaldehyde (**15**), methyl benzoate (**19**) and (dimethoxymethyl)benzene (**20**). In particular, the use of VO_x_/ZnO as a catalyst leads to the formation of benzaldehyde in 95% yield and a selectivity of 92%, while the photocatalysts VO_x_/TiO_2_ and VO_x_/NiO promote the conversion of the substrate into methyl benzoate in 94% and 54% yield, and a selectivity of 90% and 78%, respectively. Benzaldehyde (**15**) and benzoic acid (**16**) were obtained as two main products when the reaction was carried out in dichloromethane. Under these conditions using VO_x_/TiO_2_ as a catalyst, **16** was obtained as the main product in 96% yield and a selectivity of 90% (Scheme 4). 

**Figure 3:**
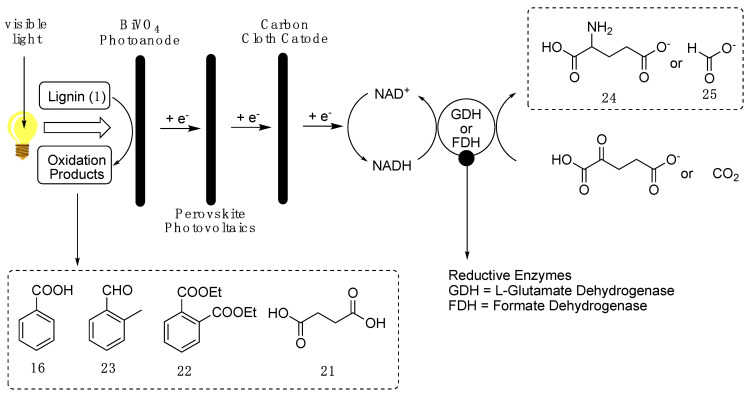
Schematic representation of the photoelectrochemical platform.

A protocol based on a combination of photochemistry and electrochemistry was recently described by Wang et al. In this work [44] a photoelectrochemical platform was developed to perform the green oxidation of lignin coupled to the green synthesis of value-added chemicals. This system consists of three different elements: a photoanode, a perovskite triple cation photovoltaic part (PV) and a carbon cloth cathode. The first component is a bismuth vanadate photoanode which oxidized lignin, the other two components were used to create a system to reduce NAD+ to NADH, that in turn acts as a cofactor of some reductive NADH-dependent enzymes such as formate dehydrogenase to reduce CO_2_ into formate (**25**), or the L-glutamate dehydrogenase to reduce α-ketoglutarate into L-glutamate (**24**). (Figure 3). 

The oxidative reaction was performed on alkali lignin and lignosulfonate lignin for 12 h and was monitored by 2D-NMR and gel permeation chromatography. Depolymerization of lignin was demonstrated by the decrease in molecular weight of the alkali lignin from 51,000 to 15,000. At the same time the NMR spectrum evidenced a breakage of the linkages with low bond dissociation energies, such as the β-O-4 linkage. It is worth mentioning, the two samples of lignin gave different results. When the oxidation was performed on lignosulfonate, the disappearance of the absorption bands of the aromatic region and an appearance of signals of the C=O stretching bands was observed by FTIR. This suggests that the oxidation is able to open the aromatic rings to carbonyl groups. On the other hand, in the case of alkali lignin, an increase in the C=O stretching bands intensity without the ring opening was detected.

Recently electrochemistry was also employed by Lan et al. [45] on cornstalk lignin resulting in the formation of different aromatic compounds. The process consists of an oxidative depolymerization at the anode that is represented by a Pb/PbO_2_ electrode and subsequently the obtained lignin fragments are reduced by the nickel cathode. Twelve different compounds were individuated as products such toluene, anisole, o-Xylene and m-Xylene, which were obtained in 36.1 g, 9.5 g, 14.4 g and 11.7 g per kg-lignin, respectively. 

**Figure 4:**
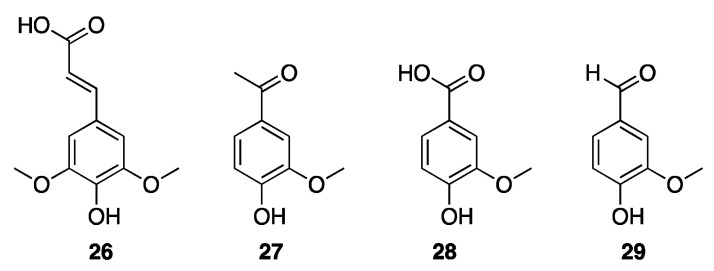
Lignin depolymerization products.

Another example in which electrochemistry was used for the lignin depolymerization was reported by Di Fidio et al. in 2021 [46]. The authors investigated the use of three different electrodes, platinum, nickel peroxide hydroxide and graphite at different pH and substrate concentrations. The technical lignin was successfully depolymerized, affording as products sinapic acid (**26**, 64.3 mg L^−1^, 0.32 wt%), acetovanillone (**27**, 30.2 mg L^−1^, 0.15 wt%), vanillic acid (**28**, 23.4 mg L^−1^, 0.12 wt%) and vanillin (**29**, 23.8 mg L^−1^, 0.12 wt%) (Figure 4).

**Scheme 5:**
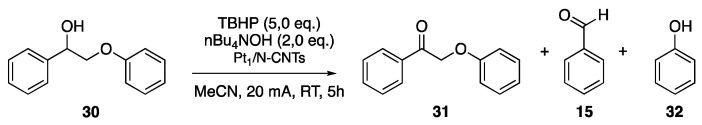
Preliminarily investigation using **30** as model compounds.

Cui et al. [47] reported an electrochemical based protocol using mild conditions and atomically dispersed Pt–N_3_C_1_ sites deposited on nitrogen-doped carbon nanotubes (Pt_1_/N-CNTs) as a catalyst. They simulated the depolymerization using Pt_1_/N-CNTs as a catalyst and 2-phenoxy-1-phenyl ethanol (**30**) as a model compound using the conditions depicted in Scheme 5. 

A mixture of ketone **31**, **15** and phenol (**32**) were obtained with a conversion of 99%. Then, the developed protocol was applied on other different lignin model compounds. In all the cases a conversion >99% was observed with benzaldehyde as the main product, demonstrating the high versatility of this method. The only exception was compound **38** that was converted only in 78% yield, probably because of the steric hindrance caused by the chlorine in meta position (Table 1).

### 2.2. Mechanochemistry in Lignin Oxidation 

**Scheme 6:**
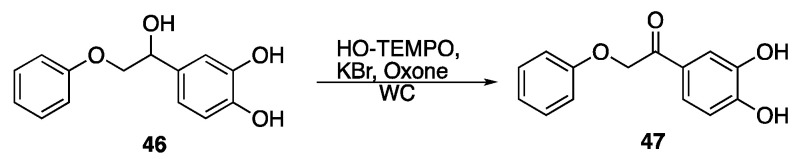
Oxidation of **46**.

Mechanochemistry represents a non-conventional technology that uses mechanical forces for the activation of chemical reactions addressing several principles of green chemistry [48,49]. The most recent examples applied to the oxidation of lignin and lignin model compounds were reported by Dabral et al. [50] in 2018 and Sun et al. [51] in 2020. The research group of Dabral pioneered the use of mechanochemistry for the degradation of lignin in 2013 [52], demonstrating that with this methodology the use of solvents and metal catalysts may be avoided. More recently [50], they reported a mechanochemically activated oxidation of lignin using Oxone^®^ as an oxidant and TEMPO or two of its derivatives (4-acetamido-TEMPO (AcNH-TEMPO) and 4-hydroxy-TEMPO (HO−TEMPO)) as catalysts. The reactions were firstly carried out on the monolignol **46** that mimic the β-O-4 linkage. The best conditions were obtained using HO−TEMPO, KBr and Oxone^®^ in ratio 0.2:0.2:1.5 and milling for 90 min at 30 Hz using as milling media tungsten carbide (WC). The corresponding ketone **47** was obtained in 97% yield a conversion of the starting material up to 99% (Scheme 6). 

The same protocol was successively applied on beechwood lignin. NMR analysis showed that after milling for 180 min with a frequency of 30 Hz the oxidation degree reached 84%, while only 6% of oxidation was obtained performing the same reaction without the catalyst. The structural changes in the lignin after oxidation were also investigated trough IR spectroscopy. A decrease in intensity of the OH bands and a subsequent increase in the C=O bands was observed. At the same time the GC-MS analyses showed 3,5-dimethoxyquinone and 2-methoxybenzoquinone as the main products. Finally, it was demonstrated that this oxidative protocol can be applied on a large scale. In fact, when the reaction was performed on 10 g of beechwood lignin, gel permeation chromatography showed a strong reduction of the molecular weight demonstrating that lignin was successfully depolymerized.

**Scheme 7:**
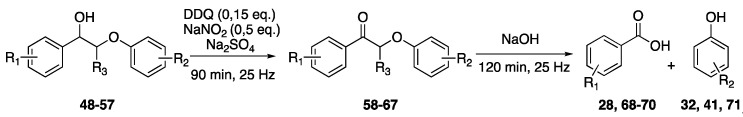
Schematic representation of the mechanochemical oxidation and depolymerization.

Another mechanochemical, solvent-free and eco-friendly lignin oxidative depolymerization protocol was developed by Sun et al. [51]. It is characterized by two oxidative steps starting from a series of lignin β-O-4 model compounds (Scheme 7 and Table 2).

The first step was performed on compounds **48**–**57**, using a DDQ/NaNO_2_ (0.15 eq/0.5 eq) as a catalytic system. In all the cases the corresponding ketones (**58**–**67**) were obtained in good yields, and they were subsequentially used as substrates for the second step. The mechanochemical depolymerization was performed using a strong base (NaOH) as a catalyst leading to the corresponding phenols (**32**, **41**, **71**) and aromatic carboxylic acids (**28**, **68**–**70**) (Table 2). This protocol was then applied on raw lignin (diaxasolv lignin) and the results showed a successful depolymerization with some selectivity in the production of syringate (**72**, 7.5 wt%). 

### 2.3. Metal Catalysts in the Lignin Oxidation 

Iron-complexes are frequently used in the oxidation of lignin and Tong et al. provided a nice example [53]. They used the low- cost Fenton catalyst consisting of Fe^3+^ that activates the green oxidant H_2_O_2,_ to oxidize lignin. The model reaction was carried out on the organosolv hardwood lignin in supercritical ethanol (7 Mpa, 250 °C). It was demonstrated that the Fenton catalyst is able to cleave the β-ether bonds through a radical process converting the lignin into organic oil, mainly composed of aromatic compounds but also dicarboxylic acids and their esters, with a yield up to 66%. 

More recently, Arefieva et al. [54] synthetized a new heterogeneous catalyst containing Fe^3+^ using plant derived silica that was obtained from rice husk. The iron-based catalyst was tested on lignin (obtained from rice husk hydrolysate), using H_2_O_2_ as an oxidant in the presence of UV and visible light irradiation. The reactions were performed using different COD (chemical oxygen demand, oxidable organic compounds: H_2_O_2_ ratio ranging from 1:2 to 1:16) and it was demonstrated that after an exposition of UV light for 15 min and then under sun light for 7 days, in the presence of the catalyst, there was a 20-fold reduction of phenols of compared to the case without a catalyst. 

Additionally, Patankar et al. in 2019 [55] used an iron-complex catalyst in the oxidation of kraft lignin performing the reaction in mild conditions and using water as a solvent. Fe@MagTEMPO is a heterogeneous catalyst that consists of TEMPO anchored in magnetic nanoparticle characterized by the presence of free amine groups. The interest on this approach is mainly focused on the facile recyclability of the catalyst that can be reused for at least five times without losing the activity.

**Scheme 8:**
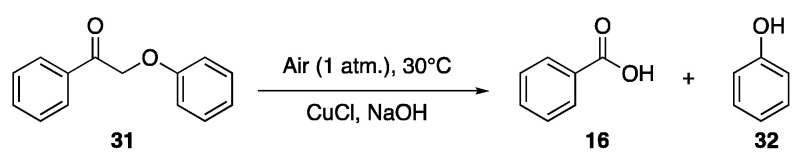
Oxidation of 2-phenoxy-1-phenylethanone.

Copper salts were also used as catalysts to selectively cleave the C-C bonds under mild basic conditions. Hu et al. [56] used compound **27** testing different bases and copper salts performing the reactions under air (1 atm) at 30 °C in water as a solvent (Scheme 8). 

The reaction carried out without a base or without a catalyst showed conversions of 0.75% and of 8.07%, respectively, demonstrating that the presence of both the elements is essential. At the same time, the effect of different bases was tested using CuCl as a catalyst. It was observed that increasing the strength of the base induces a higher conversion. With NaOH as the best base, the catalytic activity of different copper salts was investigated. In general, higher conversions were obtained using the Cu(I) salts. With the optimized conditions, a conversion of 96.96% of **31** into phenol (**32**) and benzoic acid (**16**) in 89% and 85% yield, respectively, was observed. The versatility of the protocol was demonstrated performing the reactions on different model compounds and reaching in all the cases high conversions of the substrates into the corresponding oxidized derivatives (Table 3). 

Additionally, in this case, the protocol was applied to raw lignin derivatives such as hardwood eucalyptus, softwood pine, herb corn stover, bamboo, pennisetum and bagasse performing the reactions using more drastic conditions (160 °C, 60 min, 5 bar air pressure). The results showed a very complex panel of products depending on the type of lignin used as a substrate even if, in all the cases, the main products were syringaldehyde and vanillin.

Metalloporphyrins are also an example of catalysts used for the green oxidation of lignin when the oxidant is hydrogen peroxide. These compounds are characterized by the presence of porphin pyrrole rings able to mimic the activity of various enzymes such as lignin peroxidase [57]. Artaud et al. [58] as early as in 1993 reported the use of Fe(TF_5_PP)Cl (meso-tetrakis-(pentafluorophenyl)porphyrin iron(III)) chloride as catalyst in the oxidation of 1,2 dimetoxyarenes. Some years later, the activity of metalloporphyrins immobilized on montmorillonite, was analyzed as biomimetic of lignin peroxidase. In particular, manganese meso-tetrakis(tetramethylpyridinio)porphyrinpentacetate (Mn(TmePyP)clay) immobilized onto montmorillonite was used as a catalyst. It was demonstrated that Mn(TmePyP)clay is able to convert apocinol (a lignin model compound) in 59% yield when the reaction was performed at 60 °C with reaction times from 30 min to 4 h using H_2_O_2_ as oxidant [57]. 

**Figure 5:**
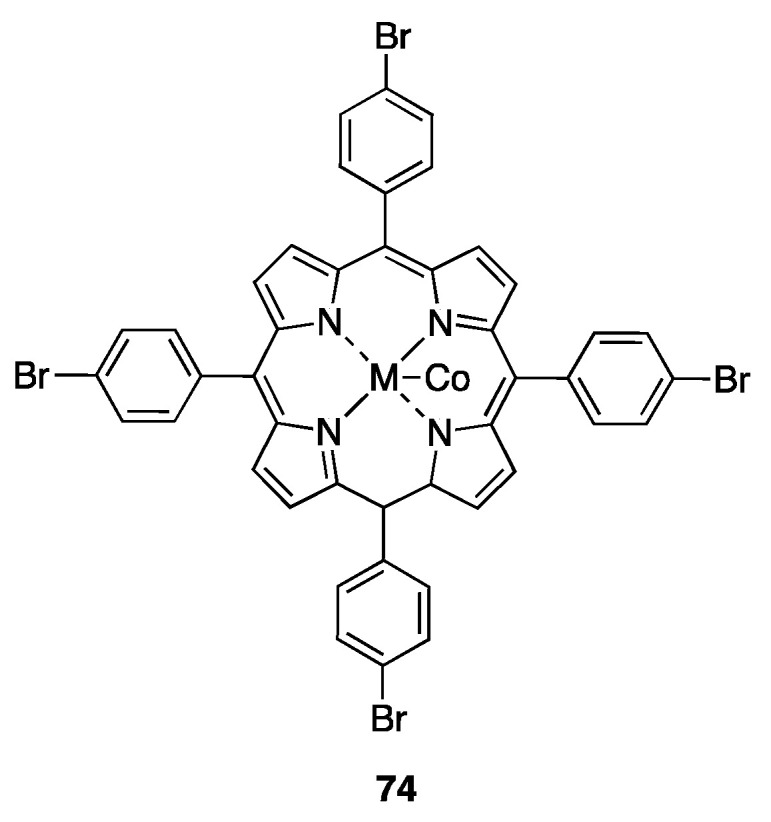
CoTBrCCl (**74**) structure.

The most recent paper on the use of the metalloporphyrins is that by Xie at al. [59]. In their work, a series of metalloporphyrins were screened and CoTBrPPCl (**74**, Figure 5) was selected as the best catalyst. This compound was tested in the degradation of lignin in the presence of H_2_O_2_ as oxidant. The results showed a good catalytic activity with a yield in aromatic compounds up to 20.1% while the reaction performed without the metalloporphyrin gave the same compounds but only in 5.6% yield.

**Figure 6:**
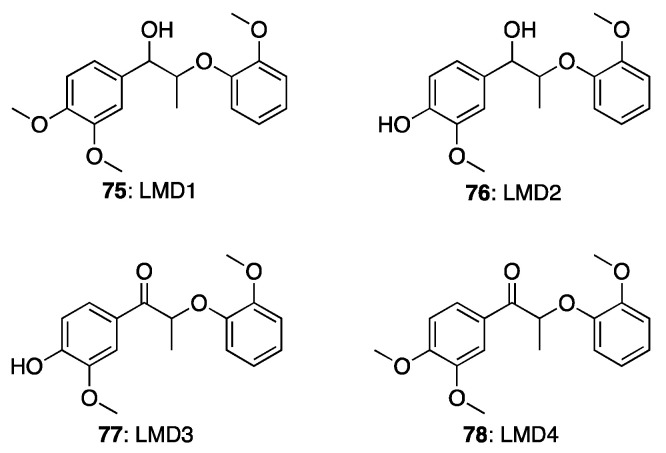
Lignin model compounds.

Phenanthroline–metal complexes were used in combination with H_2_O_2_ in alternative green oxidation processes [60]. In 2015, the catalytic activity of a 1,10 phenanthroline copper complex was investigated performing the reactions with H_2_O_2_ in basic conditions (0.05 M NaOH). Four different lignin model compounds were used as substrates (compounds **75**–**78**, Figure 6) demonstrating that phenanthroline metal complexes are able to catalyze the oxidation of the OH group in Cα position but not the β-O-4 bond cleavage [60].

In 2020, Wu et al. [61] tested the reactivity of Cu(Oac)_2_/1,10-phenanthroline in a deep eutectic solvent, methanol-choline chloride (MeOH-ChCl). MeOH-ChCl was able to improve both the solubility of alkaline lignin and the catalytic activity of phenanthroline-metal catalyst obtaining acetovanillone and acetic acid (yield 87 and 12%, respectively) as products of oxidation in 3 h at 60 °C. 

The use of polyoxometallates (POMs) as catalysts in lignin oxidation was also investigated in recent years. In 2013 the catalytic activity of H_5_Pmo_10_V_2_O_40_ was demonstrated in different substrates (pyrolytic lignin, hydrolytic lignin, alkali lignin, sodium lignosulfonate and calcium lignosulfonate). They were depolymerized using oxygen as green oxidant and leading to the formation of organic acids (dimethyl fumarate and dimethyl succinate) and aromatic compounds (aromatic aldehydes and acids). Moreover, it was also demonstrated that H_5_Pmo_10_V_2_O_40_ can be reused for five times without losing its catalytic activity [62]. 

**Scheme 9:**
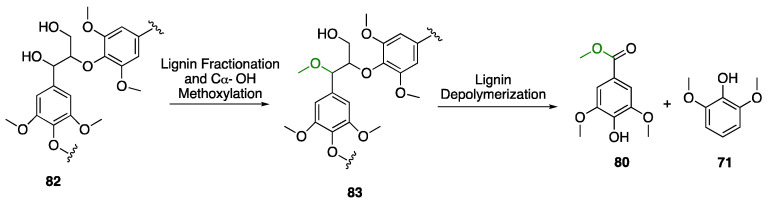
Schematic representation of lignin fractionation and depolymerization.

Du et al. [63] recently developed a new eco-sustainable lignin fractionation and oxidation method using POMs. The fractionation process consists of the removal of lignin from lignocellulosic biomass and presents various issues. The most relevant is the recondensation of lignin in acidic conditions. In their work, they demonstrated that H_3_PMo_12_O_40_ (PMo_12_) could be used as a catalyst both in preventing the condensation during the fractionation and in the lignin depolymerization. To mimic the fractionation process, different lignin model compounds were selected and subjected to the following reaction conditions: 0.25 mmol of the model compound, 0.025 mmol of PMo_12_ catalyst, 10 mL of methanol, 30 bar oxygen/nitrogen (*v*/*v* 2:28). The reactions were stirred at 100 °C for 2 h. When the reaction was carried out with 1-phenylethanol (the simplest model compound), the hydroxyl group was quantitatively converted into the methoxyl derivatives. Taking into account these preliminary data, Du et al. applied the protocol on raw lignin (Scheme 9). The fractionation step was performed using different solvents and conditions in terms of oxygen pressure, catalyst and concentration of catalysts. The best conditions were selected using a solution of methanol and water (9:1), 2.5 mmol/mL of PMO, 9 bar of oxygen/1 bar of nitrogen at 100 °C. Delignification was obtained in 96% yield and NMR analysis evidenced that the extracted lignin was etherified at the alpha position. Then, the fractionated lignin was depolymerized at 140 °C for 4 h in a mixture of methanol and water (9:1) with 10 bar O_2_/N_2_ (9:1). The GC-MS analysis evidenced that lignin was converted into low molecular weight compounds such as vanillin (**28**), methylparaben (**79**), methyl syringate (**80**) and methyl vanillate (**81**).

**Scheme 10:**
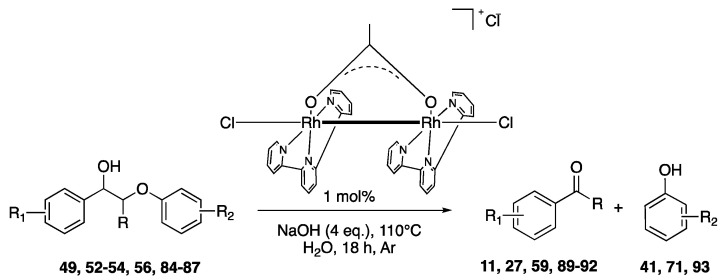
Oxidation promoted by Rh binuclear complex catalyst.

In 2019 the possibility of using binuclear Rh complexes as catalysts for oxidative depolymerization was investigated starting with the model compounds **49**. The reaction proceeds with a quantitative conversion of the substrates into the corresponding ketone **88** (86%), phenol derivative **41** (89%) and a very low quantity of the dehydrogenated intermediate **59** (8%) (Scheme 10) [64].

To demonstrate the versatility of the oxidative protocol, the optimized conditions were successively applied to different lignin model compounds. As a result, ketones (**11**, **27**, **59**, **89**–**92**) and phenols (**41**, **71**, **93**) were obtained in good yields (Table 4).

**Scheme 11:**
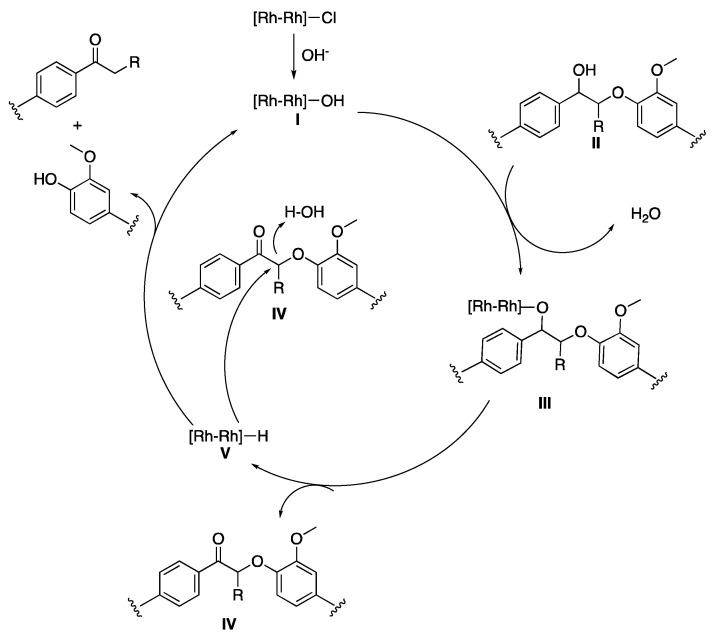
Hypothesized mechanism.

Finally, the catalytic activity of the above-mentioned Rh complex was tested on raw basswood lignin reaching a monomer yield of 2.3 wt%. From the 2D-HSQC-NMR analyses, it was demonstrated that the oxidation cleaves the linkages of the side chain and the dehydrogenation of the Cα-OH group was detected. Wang et al. hypothesized the mechanism for the cleavage of lignin (Scheme 11). Initially, because of the presence of the base (NaOH), the chloride ligand in the Rh catalyst is substituted by a hydroxide. This new complex is responsible for the deprotonation of the Cα-OH group forming intermediate **III** that undergoes β-hydride elimination to produce ketone **IV**. The Rh hydride form successively cleaves **IV** after the addition of H_2_O giving a different cleaved product and the initial form of the Rh complex (**I**) [64].

### 2.4. Metal-Free Lignin Oxidative Depolymerization

**Scheme 12:**
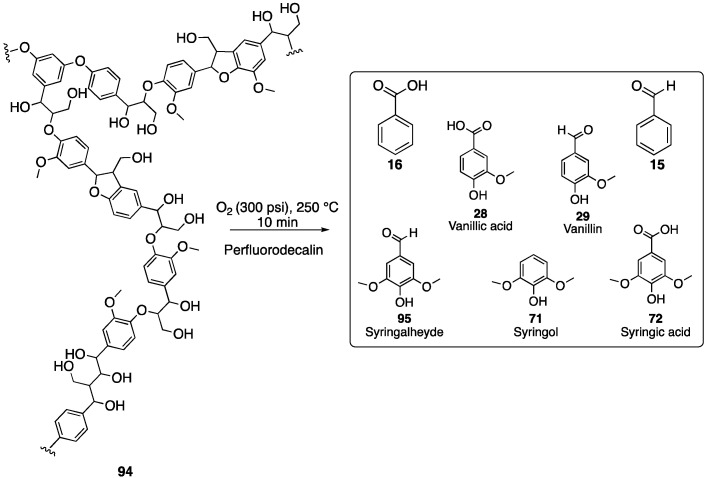
Lignin degradation in perfluorodecalin.

The oxidative depolymerization of lignin based on the use of perfluorodecalin was reported in 2020. [29] This is a solvent usually used as artificial blood due to its ability to solubilize oxygen and low toxicity. Moreover, this is a non-flammable, non-bio-accumulating and non-ozone-depleting solvent, all aspects that make perfluorodecalin an eco-friendly and “green” solvent [29]. While with classical solvents (MeOH, MeCN, But-OH, EtOAc) and 300 psi of O_2_ lignin undergoes oxidation with yields ranging from 1.0 to 2.6%, in perfluorodecalin the yield was 10.5% in phenolic monomers (Scheme 12). The same reactions were performed in oxygen-free conditions leading to no product formation, this suggests that the perfluorodecalin is able to promote the lignin oxidation because of the high oxygen solubility. Moreover, it was discovered that oxygen is mandatory not only for the oxidation, but also to avoid the condensation of lignin. In fact, when the reaction was performed in the absence of O_2_ for 20 min, the formation of oligomers was detected. In the presence of O_2_, benzaldehyde (**15**) and benzoic acid (**16**) were the main products without any condensation reaction. The authors explained this behavior stating that molecular oxygen is an inhibitor of the free radical polymerization, the process that leads to the condensation of lignin. It is worth mentioning that after a liquid–liquid separation, perfluorodecalin can be recovered without any signs of degradations.

Wang et al. [65] considered a Baeyer–Villiger (BV) reaction as a first step in the conversion of a ketone used as a lignin model compound into the corresponding aryl ester and acetal ester. The latter then undergoes alcoholysis in the presence of K_2_CO_3_. Two oxidants were used in the BV reaction: metachloroperbenzoic acid (m-CPBA) and hydrogen peroxide. When the reactions were performed with m-CPBA on substrates **31** and **73**, the corresponding acetal ester was obtained with a conversion of 100% and yield > 90%. Additionally, in the case of **17** the main product was the acetal ester with a 78% yield, while when the reactions were performed on the **58**, **63** and **96**, characterized by the presence of a methoxy group, aryl-ester was obtained as the main product. Substituting m-CPBA with hydrogen peroxide and using dibenzyldiselenide ((PhCH_2_Se)_2_) as a catalyst, similar results were obtained with the only exception of compound **17** converted in just 30% (Table 5).

The second step of this procedure consists of the alcoholysis that was carried out using K_2_CO_3_ in various alcohols: methanol, ethanol, butanol, benzyl alcohol and isopropyl alcohol affording higher yields for the less sterically hindered alcohols. 

**Scheme 13:**
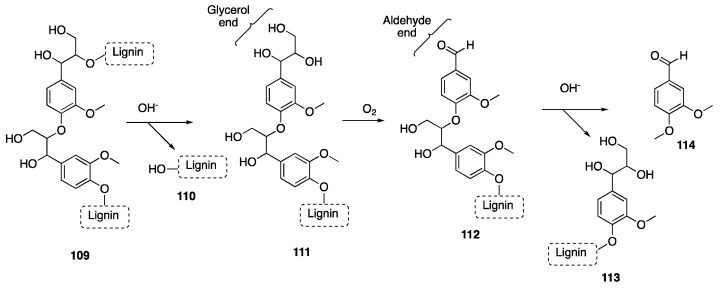
Lignin oxidation proposed mechanism.

Recently, Hosoya et al. [66] developed an aerobic oxidation in a tetrabutylammonium hydroxide (Bu_4_NOH). The reactions were performed using the conditions previously optimized by the same authors with the addition of pure oxygen [67]. Japanese cedar (*Cryptomeria japonica*) wood flour was used as substrate and the reaction was carried out at 120 °C in a Bu_4_NOH aqueous solution (1.25 mol/L) fluxing pure O_2_. After 8 h, vanillin was obtained as major product with a yield of 19.8 wt% that is higher with respect to that obtained when the reaction was performed under air (15.4 wt% yield after 43 h). The proposed mechanism is depicted in Scheme 13. The first step is the cleavage of the β-O-4 ether linkage catalyzed by strong basic conditions with the formation of the glycerol end that, in a second step, is subsequently oxidized (by oxygen) and converted into the corresponding aldehyde end. In the last step, the aldehyde end is converted into vanillin through the second cleavage of the ether linkage.

### 2.5. Selenium Catalyzed Oxidation

Selenium-catalyzed oxidative procedures are conceived as green, bioinspired transformations, because when hydrogen peroxide is the oxidant, the catalytic cycle of the key antioxidant enzyme glutathione peroxidase, is mimicked. This paradigm has been implemented by us and others in synthetic organic chemistry [68,69,70,71,72,73]. 

**Scheme 14:**
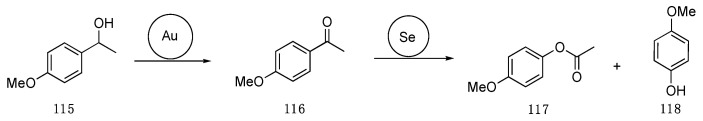
Schematic representation of two steps oxidation.

The sole example of Se-catalyzed oxidative lignin transformations has been reported by Santos et al. [74]. They proposed a novel protocol for the oxidation of 1-(4-methoxyphenyl) ethanol (**115**). The procedure starts with a gold promoted oxidation of benzyl alcohol to afford the ketone (**116**) that acts as a substrate for a subsequent BV reaction catalyzed by an organoselenium derivative (Scheme 14). Au nanoparticles were prepared reducing AuCl^4-^ with ascorbic acid and then immobilized on silica.

Initially, the reactions were performed using two different green oxidants (O_2_, H_2_O_2_) in THF both in the presence and in the absence of catalysts. From the results, it was evident that 4 mol% of gold nanoparticles are able to catalyze the oxidation of the alcohol into the ketone reaching a conversion of 99% in two hours when using H_2_O_2_ (40 mmol). To optimize the reactions, Au/SiO_2_ was tested in different solvents and the best conditions were selected when THF (conversion > 99%) and MTBE (methyl-tert-butyl ether) (conversion: 97%) were used as solvents. Then, the same reactions were performed under flow conditions, confining Au/SiO_2_ into a packed-bed reactor to enhance the recycling. Additionally, in this case different solvents and different amounts of H_2_O_2_ were tested reaching a maximum of 77% of conversion when the reaction was performed using MeOH and 40 eq of H_2_O_2_ at 40 °C. For the second step, different organoselenium compounds were evaluated as catalysts in oxidation of ketone **116**. Initially the reactions were performed testing the activity of diphenyldiselenide ((PhSe)_2_) in different solvents and in the presence of different oxidants. The results showed that the best conversion (94%) into the ester (**117**) was achieved when the reaction was carried out using methanol as a solvent and hydrogen peroxide as an oxidant. The same analyses were performed in the presence of benzenseleninic acid immobilized on a resin (PAR, Table 6). Even PAR showed the ability to convert **116** into **117** in 58%. The reactions were also carried out in continuous flow conditions. In this case, a ratio of 0.2:0.02 between the substrate and H_2_O_2_ was used with MeOH as a solvent. With a resident time of 30 min, a conversion of 91% was observed but the ester (**117**) was recovered along with phenol (**118**) and the corresponding quinone. When reducing the residence time, the yield reduced, as well as the formation of side products. 

Finally, the entire protocol was applied to 1-(4-(benzyloxy) phenyl) ethanol (**119**), 1-(4-(benzyloxy) phenyl)-2-phenyl-ethanol (**120**) and 1-(4-(benzyloxy)phenyl)-2-phenyl-propane-1,3-diol (**121**). These substrates were first oxidized with Au/SiO_2_ under continuous flow conditions, reaching 86%, 51% and 74% conversion yields, respectively. Then, the ketones **122**, **123** and **124** were used as substrates in the BV reaction and the corresponding ester and the alcohol were prepared in good yields, with the only exception of substrate **121.** In this case, the unsaturated ketone **125** was observed as a product and then was used as a substrate in the oxidative reaction, affording the corresponding esters, **126** and **127**.

## 3. Conclusions

In this review article, the most recent and green oxidative protocols for lignin depolymerization and valorization are discussed. A critical analysis highlights their current adherence to some of the 12 principles of Green Chemistry. In some cases, classic oxidants such as nitrobenzene and chlorine can be replaced with the most ecofriendly hydrogen peroxide or molecular oxygen, even if metal catalysts cannot be totally avoided. The use of an organoselenium catalyst demonstrated that it can be a convenient alternative to metal catalysts in the activation of oxygen transfer reactions from peroxides to organic substrates.

Furthermore, mechanochemistry and heterogeneous catalysis enable the reduction of wastes production of the overall processes. In the first case the use of solvents can be avoided, and in the latter, catalysts can be easily recovered, recycled and reused in subsequent reactions. 

The analysis of the results reported in the reviewed articles showed that even if the studies on model compounds are useful for the identification of some reaction mechanisms, their transability to biomass is often not totally applicable. The molecular complexity of the biomass still represents an issue deserving more investigation in order to develop protocols suitable for industrial scalability. Some of the reviewed protocols explored the scalability, demonstrating their efficiency at the level of the gram-scale [29,50]. In our opinion they could represent a good starting point to envision an effective industrial application of new green protocols for lignin treatment and valorization.

## Figures and Tables

**Table 1:** Set of tested model compounds.

Lignin Model Compounds	Conversion %	Products (Yield %)	
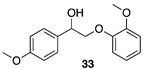	>99	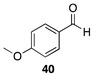	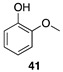
		(52%)	(31%)
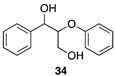	>99	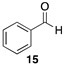	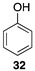
		(86%)	(65%)
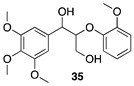	>99	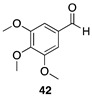	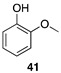
		(46%)	(23%)
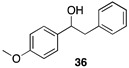	>99	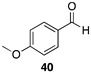	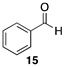
		(36%)	(34%)
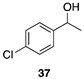	>99	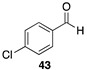	
		(78%)	
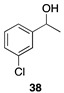	79	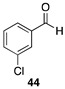	
		(59%)	
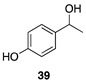	>99	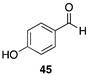	
		(36%)	

**Table 2:** Substrate scope.

First Step Product (Yield %)	Second Step Product (Yield %)	
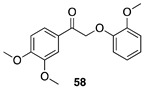	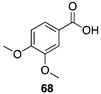	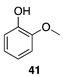
(94%)	(80%)	(95%)
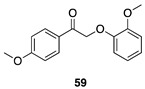	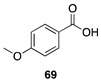	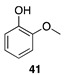
(95%)	(92%)	(99%)
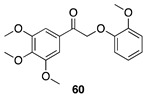	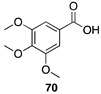	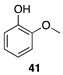
(78%)	(88%)	(98%)
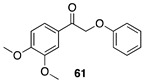	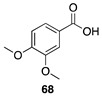	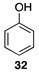
(91%)	(87%)	(96%)
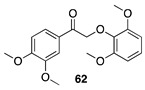	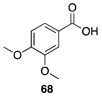	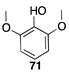
(92%)	(84%)	(88%)
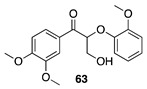	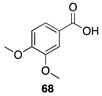	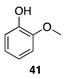
(91%)	(70%)	(92%)
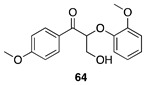	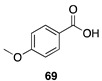	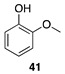
(80%)	(73%)	(90%)
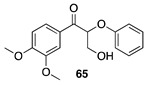	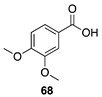	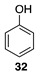
(82%)	(83%)	(96%)
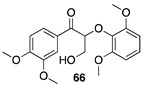	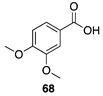	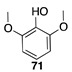
(86%)	(68%)	(87%)
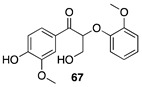	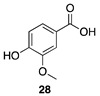	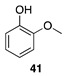
(88%)	(58%)	(92%)

**Table 3:** Scope of the reaction.

Substrates	Conditions	Products (Yields %)	
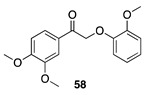	30 °C-10 h	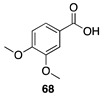	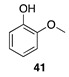
		(76.38%)	(82.59%)
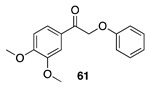	30 °C-15 h	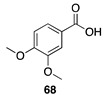	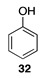
		(80.58%)	(78.23%)
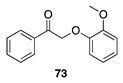	30 °C-4 h	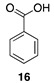	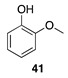
		(85.56%)	(91.28%)
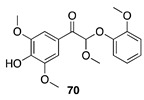	50 °C-8 h	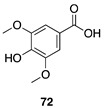	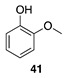
		(76.06%)	(83.57%)
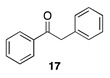	50 °C-10 h	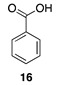	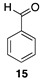
		(82.53%)	(76.65%)

**Table 4:** Substrate scope.

Substrates	Products (Yields %)		
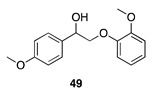	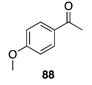	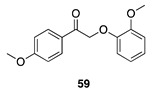	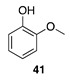
	(86%)	(8%)	(89%)
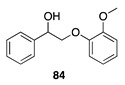	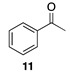		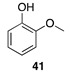
	(61%)		(86%)
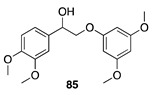	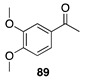		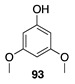
	(85%)		(90%)
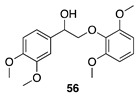	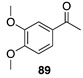		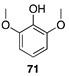
	(88%)		(92%)
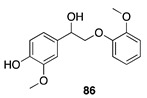	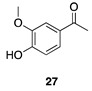		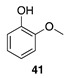
	(82%)		(86%)
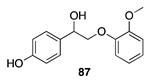	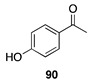		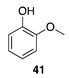
	(76%)		(90%)
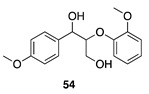	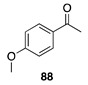	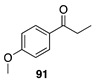	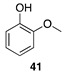
	(24%)	(34%)	(62%)
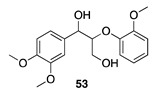	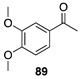	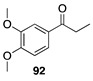	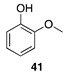
	(26%)	(33%)	(61%)
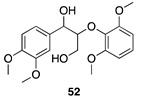	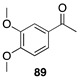	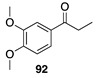	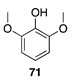
	(29%)	(32%)	(64%)

**Table 5:** BV oxidation of lignin model compounds.

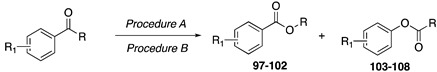						
Substrate	Conversion (%)		Products 97–102 (Yield%)		Products 103–108 (Yield%)	
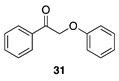	100 *^A^*	92 *^B^*	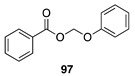		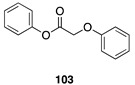	
			(99%) *^A^*	(92%) *^B^*	(0%) *^A^*	(0%) *^B^*
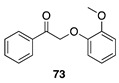	100 *^A^*	98 *^B^*	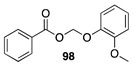		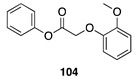	
			(92%) *^A^*	(94%) *^B^*	(0%) *^A^*	(0%) *^B^*
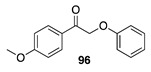	100 *^A^*	>99 *^B^*	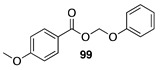		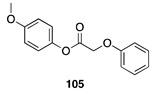	
			(6%) *^A^*	(24%) *^B^*	(90%) *^A^*	(66%) *^B^*
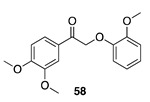	100 *^A^*	>99 *^B^*	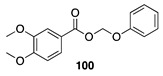		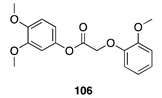	
			(3%) *^A^*	(9%) *^B^*	(86%) *^A^*	(82%) *^B^*
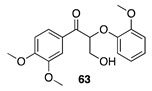	>99 *^A^*	>99 *^B^*	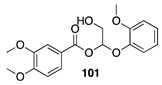		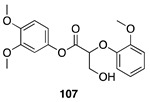	
			(26%) *^A^*	(37%) *^B^*	(66%) *^A^*	(44%) *^B^*
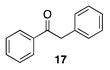	>99 *^A^*	30 *^B^*	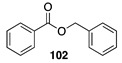		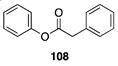	
			(78%) *^A^*	(20%) *^B^*	(7%) *^A^*	(2%) *^B^*

**Procedure A**: Substrate (0.1 mmol, 1.0 eq.), m-CPBA (0.2 mmol, 2.0 eq.), NaHCO_3_ (0.2 mmol, 2.0 eq.), 2 mL CH_2_Cl_2_, at room temperature (∼25 °C), 15 h. **Procedure B**: Substrate (0.1 mmol, 1.0 eq.), 36 wt% H_2_O_2_ (0.4 mmol, 4.0 eq.), (PhCH_2_Se)_2_ (5 mol%), 0.5 mL THF, reflux for 12 h.

**Table 6:** Lignin model compounds oxidation.

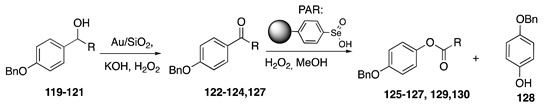			
Substrate	First Step	Second Step	
	Product (Yield%)	Product (Yield%)	Product (Yield%)
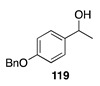	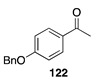	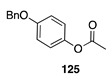	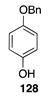
	(86%)	(13%)	(58%)
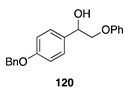	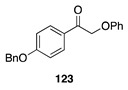	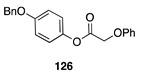	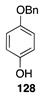
	(51%)	(40%)	(46%)
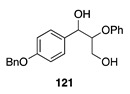	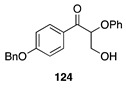	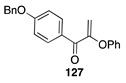	
	(74%)	(90%)	
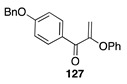		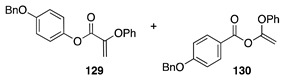	
		(25%)	
